# Bis(phenyl­sulfin­yl)methane

**DOI:** 10.1107/S1600536810005957

**Published:** 2010-02-20

**Authors:** Solange M. S. V. Wardell, Geraldo M. de Lima, James L. Wardell, Edward R. T. Tiekink

**Affiliations:** a CHEMSOL, 1 Harcourt Road, Aberdeen AB15 5NY, Scotland; b Departamento de Quimica, ICEx, Universidade Federal de Minas Gerais, 31270-901 Belo Horizonte, MG, Brazil; c Centro de Desenvolvimento Tecnológico em Saúde (CDTS), Fundação Oswaldo Cruz (FIOCRUZ), Casa Amarela, Campus de Manguinhos, Av. Brasil 4365, 21040-900, Rio de Janeiro, RJ, Brazil; d Department of Chemistry, University of Malaya, 50603 Kuala Lumpur, Malaysia

## Abstract

Two independent mol­ecules comprise the asymmetric unit of the title compound, C_13_H_12_O_2_S_2_, which differ in terms of minor variations in the relative orientations of the benzene rings [the O–S–C–C torsion angles for the first independent mol­ecule are −6.66 (17) and −12.88 (19)° compared with −21.70 (18) and 4.83 (16)° for the second mol­ecule]. Supra­molecular chains sustained by C—H⋯O contacts and aligned along the *a* axis are found in the crystal structure. These are held in place in the three dimensional structure by C—H⋯π inter­actions.

## Related literature

For the synthesis of bis­(phenyl­sulfin­yl)methane, see Shriner *et al.* (1930 ▶); Greene & Shevlin (1971 ▶); Hajipour *et al.* (2005 ▶). For separation of the *meso* and racemic forms, see Greene & Shevlin (1971 ▶). For the structure of the *meso* form, see: Kannan *et al.* (2003 ▶).
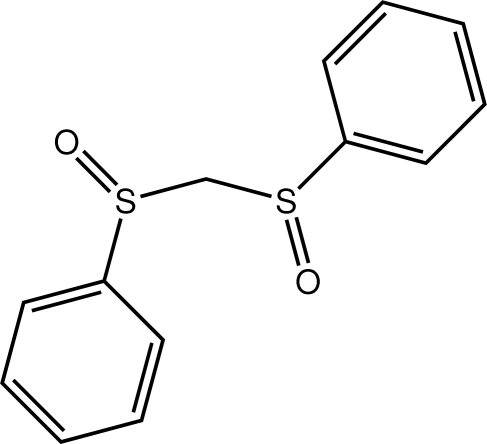


## Experimental

### 

#### Crystal data

C_13_H_12_O_2_S_2_
*M* *_r_* = 264.37
Monoclinic, 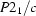

*a* = 8.4368 (4) Å
*b* = 17.1966 (7) Å
*c* = 17.1387 (6) Å
β = 95.251 (3)°
*V* = 2476.12 (18) Å^3^
*Z* = 8
Mo *K*α radiation
μ = 0.42 mm^−1^
*T* = 120 K
0.32 × 0.30 × 0.20 mm

#### Data collection

Nonius KappaCCD area-detector diffractometer
Absorption correction: multi-scan (*SADABS*; Sheldrick, 2007 ▶) *T*_min_ = 0.636, *T*_max_ = 0.746
41505 measured reflections
5503 independent reflections
4545 reflections with *I* > 2σ(*I*)
*R*_int_ = 0.045

#### Refinement

*R*[*F* ^2^ > 2σ(*F* ^2^)] = 0.039
*wR*(*F* ^2^) = 0.105
*S* = 1.02
5503 reflections
307 parameters
H-atom parameters constrained
Δρ_max_ = 0.28 e Å^−3^
Δρ_min_ = −0.40 e Å^−3^

### 

Data collection: *COLLECT* (Hooft, 1998 ▶); cell refinement: *DENZO* (Otwinowski & Minor, 1997 ▶) and *COLLECT*; data reduction: *DENZO* and *COLLECT*; program(s) used to solve structure: *SHELXS97* (Sheldrick, 2008 ▶); program(s) used to refine structure: *SHELXL97* (Sheldrick, 2008 ▶); molecular graphics: *ORTEP-3* (Farrugia, 1997 ▶) and *DIAMOND* (Brandenburg, 2006 ▶); software used to prepare material for publication: *publCIF* (Westrip, 2010 ▶).

## Supplementary Material

Crystal structure: contains datablocks general, I. DOI: 10.1107/S1600536810005957/pv2261sup1.cif

Structure factors: contains datablocks I. DOI: 10.1107/S1600536810005957/pv2261Isup2.hkl

Additional supplementary materials:  crystallographic information; 3D view; checkCIF report

## Figures and Tables

**Table 1:** Hydrogen-bond geometry (Å, °) *Cg*1 and *Cg*2 are the centroids of the C21–C26 and C2–C7 rings, respectively.

*D*—H⋯*A*	*D*—H	H⋯*A*	*D*⋯*A*	*D*—H⋯*A*
C3—H3⋯O3	0.95	2.39	3.268 (2)	153
C5—H5⋯O1^i^	0.95	2.47	3.302 (3)	146
C12—H12⋯O2^ii^	0.95	2.57	3.240 (2)	128
C22—H22⋯O2	0.95	2.35	3.285 (2)	170
C24—H24⋯O4^iii^	0.95	2.57	3.335 (2)	138
C4—H4⋯S2^i^	0.95	2.87	3.484 (2)	124
C9—H9⋯*Cg*1^iv^	0.95	2.67	3.553 (2)	154
C16—H16⋯*Cg*2^v^	0.95	2.78	3.682 (2)	159
C18—H18⋯*Cg*2^vi^	0.95	2.96	3.744 (2)	141

Symmetry codes: (i) 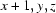; (ii) 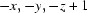; (iii) 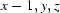; (iv) 
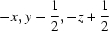
; (v) 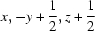; (vi) 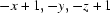.

**Fig. 1.**
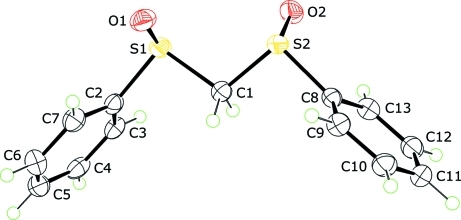
The molecular structure of the first independent molecule in (I) showing the atom-labelling scheme and displacement ellipsoids at the 50% probability level.

**Fig. 2.**
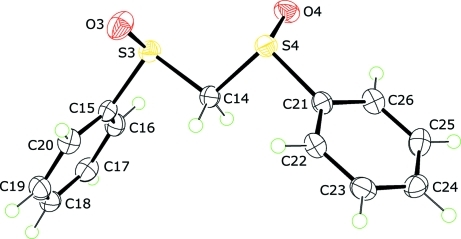
The molecular structure of the second independent molecule in (I) showing the atom-labelling scheme and displacement ellipsoids at the 50% probability level.

**Fig. 3.**
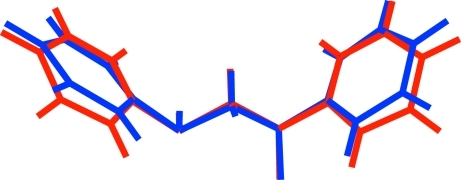
An overlay diagram highlighting the different conformations found for the independent molecules in (I). The red molecule corresponds to the molecule shown in Fig. 1.

**Fig. 4.**
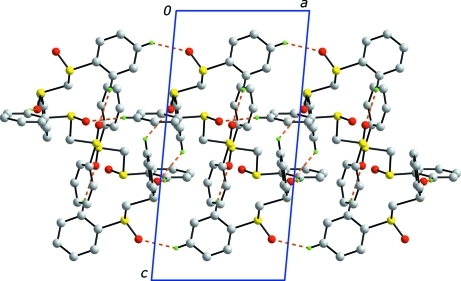
A view of a supramolecular chain aligned along the a axis in (I). The C–H···O interactions are shown as orange dashed lines. Hydrogen atoms not involved in C–H···O contacts are omitted for clarity.

**Fig. 5.**
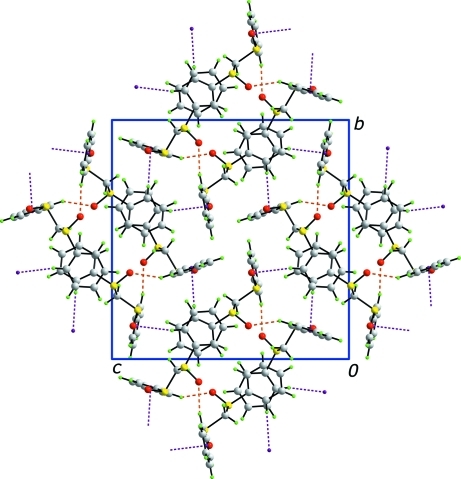
A view in projection down the a axis of the unit cell contents in (I). The two shorter of the C–H···O interactions are shown as orange dashed lines, and C–H···π interactions are shown as purple dashed lines.

## supplementary crystallographic information

### Comment

Crystals of the title compound, (I), a known species (Shriner *et al.*, 1930; Greene & Shevlin, 1971; Hajipour *et al.*, 2005), were obtained from an attempted co-crystallisation experiment (see Experimental section). Two independent molecules comprise the crystallographic asymmetric unit of (I), Figs 1 and 2. The conformations of the molecules differ only in the relative orientations of the benzene rings as illustrated in the overlay diagram, Fig. 3. These differences are quantified in terms of the O1–S1–C1–C7 and O2–S2–C8–C13 torsion angles of -6.66 (17) and -12.88 (19) °, respectively, for the first independent molecule, and for the second molecule of -21.70 (18) and 4.83 (16) °, respectively, for O3–S3–C15–C20 and O4–S4–C21–C26 torsion angles. The relative orientations of the sulfinyl groups are virtually identical as seen in the O1–S1–S2–O2 and O3–S3–S4–O4 torsion angles of -123.31 (8) and -125.32 (8) °, respectively. These are quite distinct from the equivalent value of -178.0 (1) ° found in the meso stereo-isomer (Kannan *et al.*, 2003). Otherwise, the equivalent bond distances in the three molecules show no special trends.

The crystal packing comprises supramolecular chains of molecules aligned along the *a* axis. These are sustained by C–H···O interactions as well as C–H···S and C18–H···π contacts, Fig. 4 and Table 1. Chains stack in the crystal structure being held together by C–H···π contacts, Fig. 5.

### Experimental

The title compound was prepared in accord with literature procedures (Shriner *et al.*, 1930; Greene & Shevlin, 1971; Hajipour *et al.*, 2005). The compound was isolated unchanged on slow evaporation of an ethanol solution containing equimolar (1 mmol) amounts of bis(phenylsulfinyl)methane and H_2_NCOCH_2_CH_2_SnCl_3_. The sample used in the X-ray study was further recrystallised from EtOH; m.pt. 452-454 K. Lit. value 454-456 K.(Greene & Shevlin, 1971).

### Refinement

The C-bound H atoms were geometrically placed (C–H = 0.95–0.99 Å) and refined as riding with *U_iso_*(H) = 1.2*U_eq_*(C).

### Crystal data

**Table:**

C_13_H_12_O_2_S_2_	*F*(000) = 1104
*M**_r_* = 264.37	*D*_x_ = 1.418 Mg m^−^^3^
Monoclinic, *P*2_1_/*c*	Mo *K*α radiation, λ = 0.71073 Å
Hall symbol: -P 2ybc	Cell parameters from 114673 reflections
*a* = 8.4368 (4) Å	θ = 2.9–27.5°
*b* = 17.1966 (7) Å	µ = 0.42 mm^−^^1^
*c* = 17.1387 (6) Å	*T* = 120 K
β = 95.251 (3)°	Block, colourless
*V* = 2476.12 (18) Å^3^	0.32 × 0.30 × 0.20 mm
*Z* = 8	

### Data collection

**Table:**

Nonius KappaCCD area-detector diffractometer	5503 independent reflections
Radiation source: Enraf Nonius FR591 rotating anode	4545 reflections with *I* > 2σ(*I*)
10 cm confocal mirrors	*R*_int_ = 0.045
Detector resolution: 9.091 pixels mm^-1^	θ_max_ = 27.5°, θ_min_ = 3.0°
φ and ω scans	*h* = −10→10
Absorption correction: multi-scan (*SADABS*; Sheldrick, 2007)	*k* = −22→22
*T*_min_ = 0.636, *T*_max_ = 0.746	*l* = −22→22
41505 measured reflections	

### Refinement

**Table:**

Refinement on *F*^2^	Primary atom site location: structure-invariant direct methods
Least-squares matrix: full	Secondary atom site location: difference Fourier map
*R*[*F*^2^ > 2σ(*F*^2^)] = 0.039	Hydrogen site location: inferred from neighbouring sites
*wR*(*F*^2^) = 0.105	H-atom parameters constrained
*S* = 1.02	*w* = 1/[σ^2^(*F*_o_^2^) + (0.0584*P*)^2^ + 1.1709*P*] where *P* = (*F*_o_^2^ + 2*F*_c_^2^)/3
5503 reflections	(Δ/σ)_max_ = 0.001
307 parameters	Δρ_max_ = 0.28 e Å^−^^3^
0 restraints	Δρ_min_ = −0.40 e Å^−^^3^

### Special details

**Table:**

Geometry. All s.u.'s (except the s.u. in the dihedral angle between two l.s. planes) are estimated using the full covariance matrix. The cell s.u.'s are taken into account individually in the estimation of s.u.'s in distances, angles and torsion angles; correlations between s.u.'s in cell parameters are only used when they are defined by crystal symmetry. An approximate (isotropic) treatment of cell s.u.'s is used for estimating s.u.'s involving l.s. planes.
Refinement. Refinement of *F*^2^ against ALL reflections. The weighted *R*-factor wR and goodness of fit *S* are based on *F*^2^, conventional *R*-factors *R* are based on *F*, with *F* set to zero for negative *F*^2^. The threshold expression of *F*^2^ > 2σ(*F*^2^) is used only for calculating *R*-factors(gt) etc. and is not relevant to the choice of reflections for refinement. *R*-factors based on *F*^2^ are statistically about twice as large as those based on *F*, and *R*- factors based on ALL data will be even larger.

### Fractional atomic coordinates and isotropic or equivalent isotropic displacement parameters (Å^2^)

**Table:**

	*x*	*y*	*z*	*U*_iso_*/*U*_eq_	
S1	0.24251 (6)	0.13741 (2)	0.22205 (3)	0.02771 (12)	
S2	0.04038 (6)	0.03440 (3)	0.30510 (3)	0.02665 (12)	
S3	0.49132 (6)	0.19368 (3)	0.49191 (3)	0.02721 (12)	
S4	0.28505 (6)	0.29593 (3)	0.39107 (3)	0.02590 (12)	
O1	0.12552 (17)	0.12780 (8)	0.15191 (9)	0.0349 (3)	
O2	0.02544 (18)	0.09565 (8)	0.36612 (9)	0.0376 (3)	
O3	0.49884 (19)	0.13910 (9)	0.42450 (8)	0.0386 (4)	
O4	0.39757 (17)	0.36142 (8)	0.41079 (8)	0.0333 (3)	
C1	0.2401 (2)	0.04591 (10)	0.27607 (11)	0.0264 (4)	
H1A	0.2674	0.0020	0.2425	0.032*	
H1B	0.3182	0.0475	0.3228	0.032*	
C2	0.4359 (2)	0.12423 (10)	0.18987 (11)	0.0252 (4)	
C3	0.5695 (2)	0.14283 (10)	0.24020 (11)	0.0274 (4)	
H3	0.5585	0.1599	0.2922	0.033*	
C4	0.7181 (3)	0.13606 (10)	0.21331 (12)	0.0322 (5)	
H4	0.8103	0.1479	0.2472	0.039*	
C5	0.7339 (3)	0.11204 (12)	0.13709 (13)	0.0358 (5)	
H5	0.8367	0.1079	0.1190	0.043*	
C6	0.6003 (3)	0.09403 (12)	0.08740 (12)	0.0355 (5)	
H6	0.6117	0.0773	0.0354	0.043*	
C7	0.4492 (2)	0.10045 (11)	0.11357 (11)	0.0298 (4)	
H7	0.3570	0.0887	0.0796	0.036*	
C8	0.0703 (2)	−0.05729 (10)	0.35320 (10)	0.0233 (4)	
C9	0.0409 (2)	−0.12363 (11)	0.30828 (11)	0.0276 (4)	
H9	0.0134	−0.1198	0.2534	0.033*	
C10	0.0522 (3)	−0.19562 (11)	0.34478 (12)	0.0309 (4)	
H10	0.0335	−0.2417	0.3148	0.037*	
C11	0.0907 (2)	−0.20043 (11)	0.42499 (12)	0.0315 (4)	
H11	0.0983	−0.2499	0.4498	0.038*	
C12	0.1184 (2)	−0.13364 (12)	0.46918 (11)	0.0310 (4)	
H12	0.1448	−0.1374	0.5241	0.037*	
C13	0.1076 (2)	−0.06116 (11)	0.43344 (10)	0.0267 (4)	
H13	0.1255	−0.0151	0.4635	0.032*	
C14	0.2918 (2)	0.23495 (10)	0.47830 (10)	0.0251 (4)	
H14A	0.2699	0.2664	0.5245	0.030*	
H14B	0.2113	0.1931	0.4712	0.030*	
C15	0.4548 (2)	0.13864 (10)	0.57708 (10)	0.0237 (4)	
C16	0.4973 (2)	0.17217 (11)	0.64956 (11)	0.0270 (4)	
H16	0.5487	0.2213	0.6533	0.032*	
C17	0.4632 (3)	0.13243 (12)	0.71669 (11)	0.0315 (4)	
H17	0.4891	0.1550	0.7668	0.038*	
C18	0.3916 (2)	0.06008 (12)	0.71060 (12)	0.0313 (4)	
H18	0.3671	0.0335	0.7566	0.038*	
C19	0.3554 (2)	0.02628 (11)	0.63801 (12)	0.0329 (4)	
H19	0.3087	−0.0240	0.6344	0.040*	
C20	0.3872 (2)	0.06544 (11)	0.57028 (11)	0.0288 (4)	
H20	0.3628	0.0424	0.5202	0.035*	
C21	0.0874 (2)	0.33204 (10)	0.39779 (10)	0.0231 (4)	
C22	−0.0427 (2)	0.28285 (10)	0.38143 (10)	0.0258 (4)	
H22	−0.0276	0.2294	0.3701	0.031*	
C23	−0.1946 (2)	0.31345 (11)	0.38202 (11)	0.0303 (4)	
H23	−0.2847	0.2806	0.3722	0.036*	
C24	−0.2157 (2)	0.39202 (12)	0.39694 (11)	0.0303 (4)	
H24	−0.3202	0.4127	0.3966	0.036*	
C25	−0.0852 (3)	0.44035 (11)	0.41228 (11)	0.0313 (4)	
H25	−0.1003	0.4940	0.4225	0.038*	
C26	0.0681 (2)	0.41032 (11)	0.41268 (10)	0.0275 (4)	
H26	0.1582	0.4431	0.4230	0.033*	

### Atomic displacement parameters (Å^2^)

**Table:**

	*U*^11^	*U*^22^	*U*^33^	*U*^12^	*U*^13^	*U*^23^
S1	0.0279 (3)	0.0202 (2)	0.0356 (3)	0.00118 (17)	0.0060 (2)	0.00111 (17)
S2	0.0245 (3)	0.0232 (2)	0.0328 (3)	0.00320 (17)	0.00547 (19)	0.00257 (17)
S3	0.0234 (3)	0.0320 (2)	0.0261 (2)	0.00392 (18)	0.00189 (18)	0.00415 (17)
S4	0.0247 (3)	0.0307 (2)	0.0223 (2)	0.00229 (17)	0.00193 (18)	0.00337 (17)
O1	0.0284 (8)	0.0322 (7)	0.0432 (8)	0.0031 (6)	−0.0023 (6)	0.0076 (6)
O2	0.0444 (9)	0.0238 (7)	0.0476 (8)	0.0039 (6)	0.0212 (7)	−0.0038 (6)
O3	0.0416 (9)	0.0494 (9)	0.0247 (7)	0.0163 (7)	0.0033 (6)	−0.0021 (6)
O4	0.0245 (8)	0.0385 (8)	0.0367 (8)	−0.0059 (6)	0.0008 (6)	0.0093 (6)
C1	0.0264 (11)	0.0237 (9)	0.0294 (9)	0.0006 (7)	0.0038 (8)	0.0020 (7)
C2	0.0281 (11)	0.0193 (8)	0.0281 (9)	−0.0013 (7)	0.0031 (7)	0.0043 (7)
C3	0.0320 (11)	0.0216 (9)	0.0276 (9)	−0.0030 (7)	−0.0022 (8)	0.0045 (7)
C4	0.0296 (12)	0.0230 (9)	0.0421 (11)	−0.0034 (7)	−0.0062 (9)	0.0094 (8)
C5	0.0267 (12)	0.0346 (11)	0.0474 (12)	0.0005 (8)	0.0105 (9)	0.0100 (9)
C6	0.0363 (13)	0.0401 (11)	0.0314 (10)	0.0000 (9)	0.0098 (9)	0.0014 (8)
C7	0.0313 (12)	0.0296 (10)	0.0276 (9)	−0.0019 (8)	−0.0018 (8)	0.0019 (7)
C8	0.0196 (10)	0.0242 (8)	0.0264 (9)	−0.0002 (7)	0.0033 (7)	0.0004 (7)
C9	0.0301 (11)	0.0277 (9)	0.0248 (9)	−0.0001 (8)	0.0010 (8)	−0.0024 (7)
C10	0.0325 (12)	0.0240 (9)	0.0366 (10)	−0.0004 (8)	0.0052 (8)	−0.0038 (7)
C11	0.0302 (12)	0.0281 (10)	0.0370 (11)	0.0028 (8)	0.0077 (8)	0.0080 (8)
C12	0.0270 (11)	0.0410 (11)	0.0249 (9)	0.0045 (8)	0.0025 (8)	0.0039 (8)
C13	0.0244 (10)	0.0295 (9)	0.0259 (9)	−0.0010 (7)	0.0008 (7)	−0.0052 (7)
C14	0.0223 (10)	0.0270 (9)	0.0258 (9)	0.0029 (7)	0.0010 (7)	0.0042 (7)
C15	0.0210 (10)	0.0241 (9)	0.0257 (9)	0.0049 (7)	0.0007 (7)	0.0001 (7)
C16	0.0273 (11)	0.0232 (9)	0.0300 (9)	0.0006 (7)	0.0002 (8)	−0.0035 (7)
C17	0.0320 (12)	0.0378 (11)	0.0243 (9)	0.0064 (8)	0.0005 (8)	−0.0028 (8)
C18	0.0273 (11)	0.0347 (10)	0.0329 (10)	0.0075 (8)	0.0072 (8)	0.0097 (8)
C19	0.0289 (11)	0.0252 (9)	0.0447 (12)	−0.0017 (8)	0.0034 (9)	0.0032 (8)
C20	0.0268 (11)	0.0283 (9)	0.0304 (10)	0.0004 (8)	−0.0029 (8)	−0.0038 (7)
C21	0.0255 (10)	0.0248 (9)	0.0184 (8)	0.0007 (7)	−0.0010 (7)	0.0024 (6)
C22	0.0303 (11)	0.0225 (8)	0.0241 (9)	−0.0015 (7)	−0.0008 (7)	0.0025 (7)
C23	0.0268 (11)	0.0352 (10)	0.0284 (10)	−0.0055 (8)	0.0004 (8)	0.0073 (8)
C24	0.0267 (11)	0.0401 (11)	0.0245 (9)	0.0070 (8)	0.0047 (8)	0.0043 (8)
C25	0.0365 (12)	0.0291 (9)	0.0276 (9)	0.0075 (8)	−0.0010 (8)	−0.0021 (7)
C26	0.0324 (11)	0.0249 (9)	0.0245 (9)	−0.0019 (8)	−0.0022 (8)	−0.0005 (7)

### Geometric parameters (Å, °)

**Table:**

S1—O1	1.4931 (15)	C10—H10	0.9500
S1—C2	1.784 (2)	C11—C12	1.384 (3)
S1—C1	1.8268 (18)	C11—H11	0.9500
S2—O2	1.4978 (14)	C12—C13	1.388 (3)
S2—C8	1.7864 (18)	C12—H12	0.9500
S2—C1	1.811 (2)	C13—H13	0.9500
S3—O3	1.4945 (14)	C14—H14A	0.9900
S3—C15	1.7898 (18)	C14—H14B	0.9900
S3—C14	1.8220 (19)	C15—C20	1.383 (3)
S4—O4	1.4917 (14)	C15—C16	1.386 (3)
S4—C21	1.7928 (19)	C16—C17	1.391 (3)
S4—C14	1.8225 (18)	C16—H16	0.9500
C1—H1A	0.9900	C17—C18	1.383 (3)
C1—H1B	0.9900	C17—H17	0.9500
C2—C7	1.385 (3)	C18—C19	1.382 (3)
C2—C3	1.393 (3)	C18—H18	0.9500
C3—C4	1.380 (3)	C19—C20	1.389 (3)
C3—H3	0.9500	C19—H19	0.9500
C4—C5	1.388 (3)	C20—H20	0.9500
C4—H4	0.9500	C21—C26	1.383 (3)
C5—C6	1.384 (3)	C21—C22	1.394 (3)
C5—H5	0.9500	C22—C23	1.387 (3)
C6—C7	1.394 (3)	C22—H22	0.9500
C6—H6	0.9500	C23—C24	1.390 (3)
C7—H7	0.9500	C23—H23	0.9500
C8—C13	1.384 (2)	C24—C25	1.385 (3)
C8—C9	1.386 (2)	C24—H24	0.9500
C9—C10	1.386 (3)	C25—C26	1.392 (3)
C9—H9	0.9500	C25—H25	0.9500
C10—C11	1.386 (3)	C26—H26	0.9500
			
O1—S1—C2	107.03 (9)	C11—C12—C13	120.18 (17)
O1—S1—C1	105.99 (8)	C11—C12—H12	119.9
C2—S1—C1	95.76 (8)	C13—C12—H12	119.9
O2—S2—C8	108.42 (8)	C8—C13—C12	118.78 (17)
O2—S2—C1	104.77 (9)	C8—C13—H13	120.6
C8—S2—C1	97.34 (8)	C12—C13—H13	120.6
O3—S3—C15	108.72 (8)	S3—C14—S4	106.76 (10)
O3—S3—C14	104.46 (8)	S3—C14—H14A	110.4
C15—S3—C14	94.86 (8)	S4—C14—H14A	110.4
O4—S4—C21	107.47 (8)	S3—C14—H14B	110.4
O4—S4—C14	106.09 (8)	S4—C14—H14B	110.4
C21—S4—C14	96.09 (8)	H14A—C14—H14B	108.6
S2—C1—S1	106.69 (10)	C20—C15—C16	121.66 (17)
S2—C1—H1A	110.4	C20—C15—S3	120.88 (14)
S1—C1—H1A	110.4	C16—C15—S3	117.46 (14)
S2—C1—H1B	110.4	C15—C16—C17	118.74 (17)
S1—C1—H1B	110.4	C15—C16—H16	120.6
H1A—C1—H1B	108.6	C17—C16—H16	120.6
C7—C2—C3	121.43 (18)	C18—C17—C16	120.11 (18)
C7—C2—S1	119.05 (15)	C18—C17—H17	119.9
C3—C2—S1	119.39 (15)	C16—C17—H17	119.9
C4—C3—C2	118.86 (18)	C19—C18—C17	120.36 (18)
C4—C3—H3	120.6	C19—C18—H18	119.8
C2—C3—H3	120.6	C17—C18—H18	119.8
C3—C4—C5	120.52 (19)	C18—C19—C20	120.32 (18)
C3—C4—H4	119.7	C18—C19—H19	119.8
C5—C4—H4	119.7	C20—C19—H19	119.8
C6—C5—C4	120.2 (2)	C15—C20—C19	118.73 (17)
C6—C5—H5	119.9	C15—C20—H20	120.6
C4—C5—H5	119.9	C19—C20—H20	120.6
C5—C6—C7	120.09 (19)	C26—C21—C22	121.60 (18)
C5—C6—H6	120.0	C26—C21—S4	118.37 (14)
C7—C6—H6	120.0	C22—C21—S4	119.80 (14)
C2—C7—C6	118.90 (18)	C23—C22—C21	118.68 (17)
C2—C7—H7	120.5	C23—C22—H22	120.7
C6—C7—H7	120.5	C21—C22—H22	120.7
C13—C8—C9	121.68 (17)	C22—C23—C24	120.29 (18)
C13—C8—S2	120.71 (14)	C22—C23—H23	119.9
C9—C8—S2	117.37 (14)	C24—C23—H23	119.9
C8—C9—C10	118.88 (17)	C23—C24—C25	120.35 (19)
C8—C9—H9	120.6	C23—C24—H24	119.8
C10—C9—H9	120.6	C25—C24—H24	119.8
C11—C10—C9	120.08 (17)	C24—C25—C26	120.03 (18)
C11—C10—H10	120.0	C24—C25—H25	120.0
C9—C10—H10	120.0	C26—C25—H25	120.0
C10—C11—C12	120.39 (17)	C21—C26—C25	119.03 (18)
C10—C11—H11	119.8	C21—C26—H26	120.5
C12—C11—H11	119.8	C25—C26—H26	120.5
			
O2—S2—C1—S1	−69.82 (11)	O3—S3—C14—S4	−68.19 (11)
C8—S2—C1—S1	178.88 (9)	C15—S3—C14—S4	−178.96 (9)
O1—S1—C1—S2	−61.94 (11)	O4—S4—C14—S3	−65.87 (11)
C2—S1—C1—S2	−171.52 (10)	C21—S4—C14—S3	−176.05 (9)
O1—S1—C2—C7	−6.66 (17)	O3—S3—C15—C20	−21.70 (18)
C1—S1—C2—C7	102.03 (15)	C14—S3—C15—C20	85.36 (17)
O1—S1—C2—C3	169.22 (13)	O3—S3—C15—C16	158.16 (15)
C1—S1—C2—C3	−82.09 (15)	C14—S3—C15—C16	−94.78 (16)
C7—C2—C3—C4	−1.0 (3)	C20—C15—C16—C17	−3.3 (3)
S1—C2—C3—C4	−176.81 (13)	S3—C15—C16—C17	176.85 (15)
C2—C3—C4—C5	0.8 (3)	C15—C16—C17—C18	1.5 (3)
C3—C4—C5—C6	−0.5 (3)	C16—C17—C18—C19	0.9 (3)
C4—C5—C6—C7	0.4 (3)	C17—C18—C19—C20	−1.6 (3)
C3—C2—C7—C6	1.0 (3)	C16—C15—C20—C19	2.7 (3)
S1—C2—C7—C6	176.77 (15)	S3—C15—C20—C19	−177.49 (15)
C5—C6—C7—C2	−0.7 (3)	C18—C19—C20—C15	−0.2 (3)
O2—S2—C8—C13	−12.88 (19)	O4—S4—C21—C26	4.83 (16)
C1—S2—C8—C13	95.40 (17)	C14—S4—C21—C26	113.84 (14)
O2—S2—C8—C9	161.56 (15)	O4—S4—C21—C22	179.36 (13)
C1—S2—C8—C9	−90.15 (16)	C14—S4—C21—C22	−71.63 (15)
C13—C8—C9—C10	−1.2 (3)	C26—C21—C22—C23	−1.5 (3)
S2—C8—C9—C10	−175.59 (15)	S4—C21—C22—C23	−175.83 (13)
C8—C9—C10—C11	0.6 (3)	C21—C22—C23—C24	1.4 (3)
C9—C10—C11—C12	−0.1 (3)	C22—C23—C24—C25	−0.8 (3)
C10—C11—C12—C13	0.0 (3)	C23—C24—C25—C26	0.1 (3)
C9—C8—C13—C12	1.2 (3)	C22—C21—C26—C25	0.8 (3)
S2—C8—C13—C12	175.36 (15)	S4—C21—C26—C25	175.27 (14)
C11—C12—C13—C8	−0.5 (3)	C24—C25—C26—C21	−0.1 (3)

### Hydrogen-bond geometry (Å, °)

**Table:**

Cg1 and Cg2 are the centroids of the C21–C26 and C2–C7 rings, respectively.

**Table:**

*D*—H···*A*	*D*—H	H···*A*	*D*···*A*	*D*—H···*A*
C3—H3···O3	0.95	2.39	3.268 (2)	153
C5—H5···O1^i^	0.95	2.47	3.302 (3)	146
C12—H12···O2^ii^	0.95	2.57	3.240 (2)	128
C22—H22···O2	0.95	2.35	3.285 (2)	170
C24—H24···O4^iii^	0.95	2.57	3.335 (2)	138
C4—H4···S2^i^	0.95	2.87	3.484 (2)	124
C9—H9···Cg1^iv^	0.95	2.67	3.553 (2)	154
C16—H16···Cg2^v^	0.95	2.78	3.682 (2)	159
C18—H18···Cg2^vi^	0.95	2.96	3.744 (2)	141

Symmetry codes:  (i) *x*+1, *y*, *z*; (ii) −*x*, −*y*, −*z*+1; (iii) *x*−1, *y*, *z*; (iv) −*x*, *y*−1/2, −*z*+1/2; (v) *x*, −*y*+1/2, *z*+1/2; (vi) −*x*+1, −*y*, −*z*+1.
